# Prospective study of efficacy and safety of non-ablative 1927 nm fractional thulium fiber laser in Asian skin photoaging

**DOI:** 10.3389/fsurg.2023.1076848

**Published:** 2023-03-06

**Authors:** Xianwen Li, Si Qin, Siman Shi, Yingjun Feng, Huarun Li, Yuqin Feng, Mujin Li, Ju Wen

**Affiliations:** ^1^ The Second School of Clinical Medicine, Southern Medical University, Guangzhou, China; ^2^ Department of Dermatology, Guangdong Second Provincial General Hospital, Guangzhou, China

**Keywords:** 1927 nm thulium fiber laser, non-ablative fractional laser, photoaging, wrinkles, pores, high-frequency ultrasound

## Abstract

**Background and Objective:**

Photoaging manifests as deeper wrinkles and larger pores. It has been tried to rejuvenate photoaging skin using a variety of lasers, including fractionated lasers, which are a popular photorejuvenation treatment. A new breakthrough for skin rejuvenation is the 1927 nm fractional thulium fiber laser (FTL), a laser and light-based treatment option. Clinical data regarding the FTL for treating photoaging are limited despite its effectiveness and safety. This study is aim to evaluate FTL' clinical effectiveness and safety.

**Methods:**

Fitzpatrick skin types II–IV subjects with mild to moderate photoaging signs were enrolled in this prospective study. At intervals of one month, patients received three full face treatments. Wrinkles, spots, texture, pores, melanin index, erythema index (MI and EI), skin elasticity and hydration were measured with non-invasive tool. The epidermal thickness and dermal density on ultrasonography were compared between baseline and one month after all treatment sessions. The Global Score for Photoaging scale (GSP) was rated by two independent evaluators at the baseline and final follow-up visit. Secondary outcomes included patient-rated pain on a 10-point visual analog scale (VAS), as well as overall satisfaction. Following each treatment, adverse events were noted.

**Results:**

Totally 27 subjects (24 females and 3 males) with Fitzpatrick skin types II to IV and a mean age of 44.41 (range33–64) were enrolled. Results suggests that the epidermal thickness has significantly improved after treatment. Statistically significant improvements in melanin index, skin elasticity and wrinkles were noted. An analysis of 12 subjects' reports (44%) suggested their skin felt brighter. No post-inflammatory hyperpigmentation changes or adverse events were observed. 70% patients reporting “satisfied” or “extremely satisfied”.

**Conclusions:**

In this study, FTL was found to be a safe and effective treatment option for treating photoaging.

## 1. Introduction

The skin is the largest organ of the human body, and skin aging is a complex biological process, it results from both the intrinsic and chronological factors and from cumulative exposure to external factors (extrinsic aging) (1). The extrinsic aging is defined as premature skin aging, which is also known as skin photoaging, caused by chronic exposure to ultraviolet radiation (2). Clinical signs of photoaging include fine and deep wrinkles, larger pore size, changes in pigmentation, loss of elasticity and laxity, epidermal thinning and telangiectasia dyspigmentation (3–5).

Photoaging accounts for 80% of skin extrinsic aging (6). At advanced stages of sun exposure could increase the risk of developing actinic keratosis (AK) and skin cancer (7, 8).

Skin photoaging is of aesthetic concern worldwide. With an aging demographic shift, the demand for safe and effective treatments for photoaging has increased steadily over the last several decades. To counteract, prevent or treat photoaging, many methods are available, such as over-the-counter topicals (9), chemical peels (10) and laser therapy (11).

Fractional photothermolysis (FP) was introduced to overcome the limitations posed by conventional lasers. This kind of laser system extremely reducing the risk of side effects (12, 13). The use of fractional laser in photoaging began with ablative fractional carbon dioxide laser (14). Moreover, non-ablative fractional laser (NAFL) is one of the laser options frequently used by aesthetic dermatologists. The NAFL uses mid-infrared lasers with wavelengths ranging from 1,320 to 1940 nm to target water as a chromophore for facial rejuvenation. For over 15 years, this treatment modality has demonstrated safety, efficacy and versatility (15).

Particularly, the 1927 nm fractional thulium fiber laser (FTL) reduces the superficial signs of photoaging such as dyspigmentation, wrinkles and actinic damage (16, 17). Although there are reports that the FTL is effective and safe for treating skin conditions, clinical data regarding this modality are limited.

In this study, we aimed to investigate the efficacy and safety of FTL improving photoaging on Chinese facial skin.

## 2. Materials and methods

### 2.1. Study design and patient characteristics

This is a prospective clinical trial to investigate the efficacy and safety of FTL (Lavieen, Korea) in the treatment of photoaging patients. This study was reviewed and approved by the ethic committee of Guangdong Second Provincial General Hospital (2021-KZ-154-03) and conformed to the guidelines of the 1975 Declaration of Helsinki. All study subjects provided written informed consent before the study began. This clinical study was registered at Chinese Clinical Trial Registry with registration number ChiCTR2100050801. Inclusion criteria were healthy Chinses male and female subjects aged 30–65 with Fitzpatrick skin types II–IV, with baseline presence of moderate to severe photodamage. The severe photodamage was evaluated by GSP scale. Criteria for exclusion include the following: (1) There was infection in the treatment site; (2) had a propensity for keloid forming; (3) received chemical peeling or laser treatment in the past six months; (4) received botulinum toxin type A injection and soft tissue fillers during the previous six months; (5) received oral isotretinoin six month before the study; (6) psychopathological syndromes such as depression, anxiety, psychoticism etc.; (7) pregnancy or breast-feeding; (8) took any skin anti-aging measures and who are being treated for other skin conditions during the study time.

### 2.2. Intervention

Each subject received three treatments at 30 (+10)-day intervals. The appropriate skin area was cleansed with a mild cleanser before treatment. A topical anesthetic cream (lidocaine 2.5%/prilocaine 2.5%) was applied to the treatment area for 40–60 min under occlusion before laser treatment. The treatments were all performed by a single investigator who did not evaluate the results. The parameters were selected according to the skin condition of each patient. The laser was set to Random Mode and the specific parameters were controlled as follows: output power of 6–9 W, the pulse duration was 500–800 us and 1–2 passes performed. The endpoint was mild to moderate diffuse erythema. Immediately following treatment, the application of anti-inflammatory dressing and a cooling pack was suggested. Subjects were advised to avoid exposure to sunlight, trauma or friction.

### 2.3. Investigator-evaluated outcomes

The objective efficacy evaluation was based on standardized digital pictures, skin clinical indicators and skin ultrasound at baseline and each follow-up. A facial skin imaging analyzer, VISIA (Canfield Scientific, USA), was used to take standard digital photographs at baseline, the moment before the second treatment (M1), the moment before the third treatment (M2) and one month after the final treatment (M3). In addition, the epidermal thickness and dermal density was measured with a high frequency ultrasound scanner (DUB skinScanner, Germany). The melanin and erythema index (MI and EI), skin elasticity and hydration were measured with the high frequency skin ultrasonography (Dermascan C USB, Denmark). The degree of photoaging and the efficacy of treatment were evaluated using Global Scores for Photoaging (GSP) scale (18) by two dermatologists who performed blinded clinical assessments.

### 2.4. Patient-evaluated outcomes and safety

Subjective evaluation contained the degree of pain on a visual analog scale (VAS) and subjective satisfaction score rated by patients. At the end of the study, subjects rated their overall satisfaction on a 4-point scale, 0–3, (3 = extremely satisfied, 2 = satisfied, 1 = a little satisfied, 0 = totally dissatisfied).

After each treatment visit, treatment-related adverse reactions were evaluated including erythema, edema, crusts, erosions, stinging, burning sensations, bleeding, numbness, itching and dryness.

### 2.5. Statistical analysis

Statistical evaluation was performed with GraphPad Prism eversion 8.0, (GraphPad Software, San Diego, CA, USA). All statistical tests were two-sided and performed at a 5% confidence interval. The paired Student's t-test was used to determine the difference in mean between groups. All data are represented as mean ± standard error of the mean (SEM).

## 3. Result

31 subjects with Fitzpatrick skin types (FSTs) II-IV were enrolled in this study. 4 subjects were lost to follow-up, therefore they were excluded from the final analysis. A total of 27 patients [FSTs II (5/27, 18.5%), III (16/27, 59.2%) and IV (6/27, 22.2%)] with 3–4 GSP score completed all follow-up assessments. Using medical photographs as a basis for evaluation, a five-point global score scale (GSP) was calculated for the clinical responses. Patients were predominantly female [3 (11.1%) males and 24 (88.9%) females] with a mean age of 44.4 ± 7.71 (range33–64). Patient demographics are summarized in Table 1.

**Table 1:** Patient demographics.

Characteristics	Number of cases (%)
Patients	27
**Gender**
Male	3 (11.1%)
Female	24 (88.9%)
Mean age (SD, min-max)	44.4 ± 7.71(33–64)
Fitzpatrick skin types, *n* (%)	
II	5(18.5%)
III	16 (59.2%)
IV	6 (22.2%)
Global score for *p*hotoaging	3.44 ± 0.50

### 3.1. Objective evaluation

#### 3.1.1. Measurements of epidermal thickness and dermal density

Changes in epidermal thickness and dermal density at different time points after each treatment were detected using high-frequency ultrasonography scanner. The researchers obtained readings from 4 cm outsight the right corner of mouth region and analyzed the results. One month after three treatments, there was significantly improved in epidermal thickness compared to baseline show by the high-frequency ultrasonography scanner (Table 2). An increase in dermis density was observed, yet the differences were not significant.

**Table 2:** Comparison of epidermal thickness and dermal density before and after treatment.

Objective Score		Mean (SD)	*P*-value*
Epidermal thickness	Baseline	0.156 ± 0.0073	
	M1	0.157 ± 0.0068	0.153
	M3	0.158 ± 0.0075	0.046**
Dermal density	Baseline	27.86 ± 13.23	
	M1	30.39 ± 9.879	0.209
	M3	30.90 ± 11.38	0.729

* Epidermal Thickness or Dermal Density compared to baseline.

** Statistical significance defined as *p *< 0.05.

#### 3.1.2. Measured skin parameters

**Figure 1:**
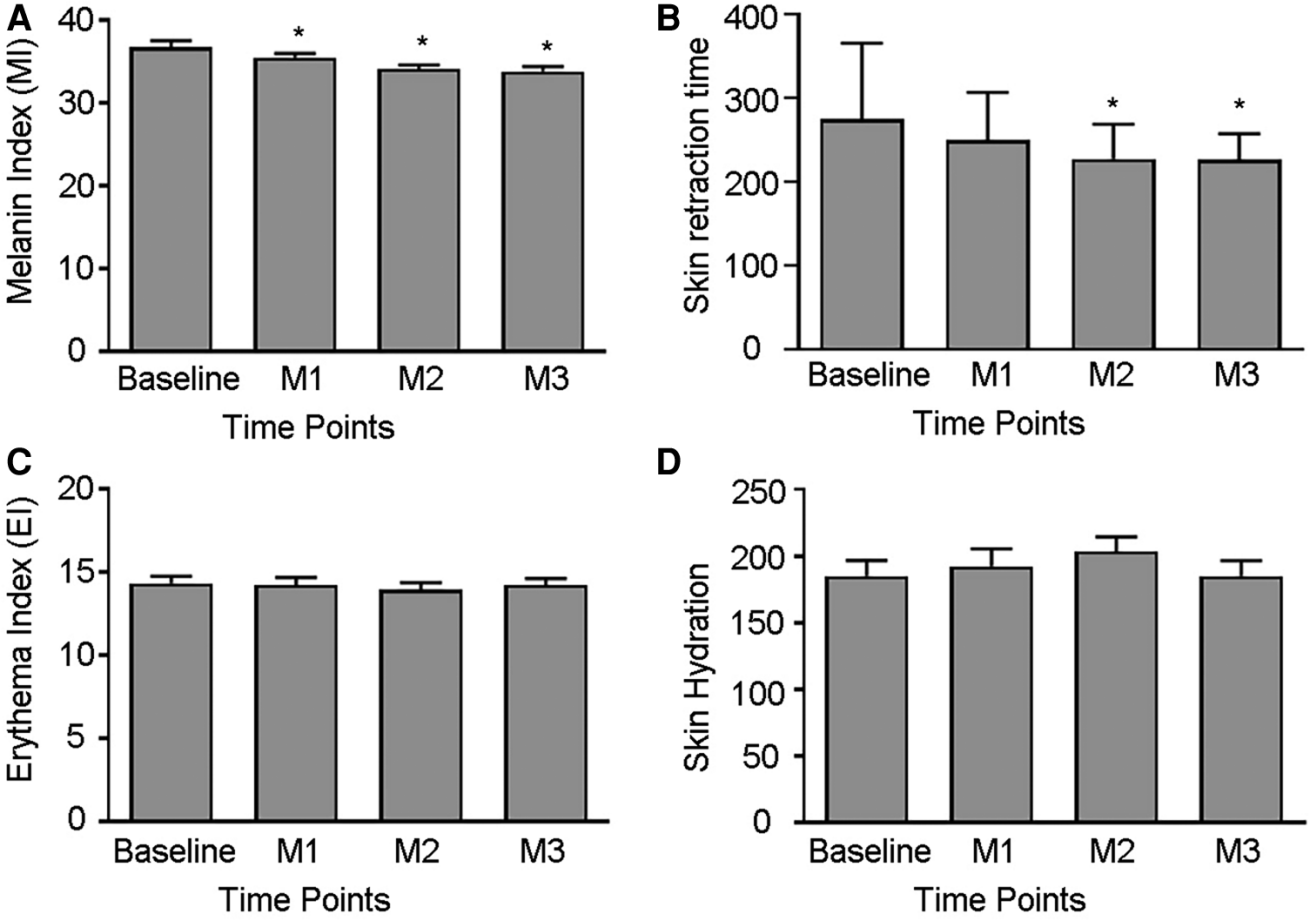
Measured skin parameters. (**A–D**) Comparison of Melanin Index, Skin retraction time, Erythema Index and Skin Hydration before and after treatment. (*p < 0.05 vs. Baseline).

The results of this study showed that FTL could result in statistically significant improvements in MI after first treatment and a statistically significant improvement in skin elasticity after second session compare with baseline. There were not significance improvements shown on the hydration and EI after three treatments (Figure 1).

#### 3.1.3. Measurements of VISIA

**Figure 2:**
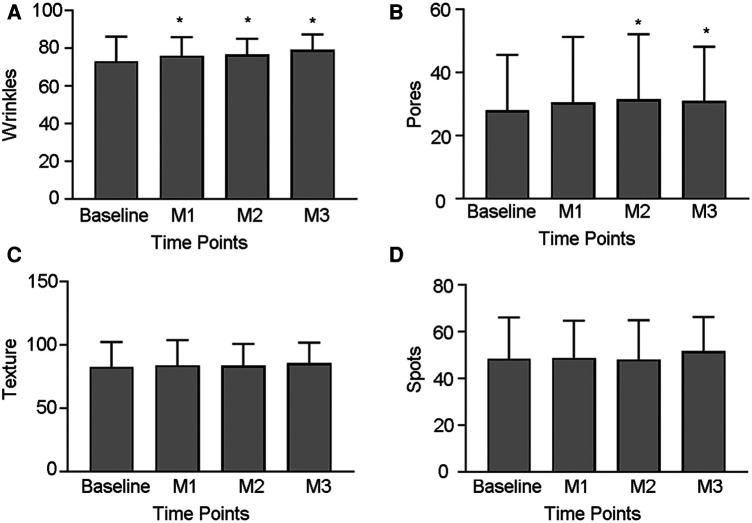
Measurements of VISIA. (**A–D**) Comparison of wrinkles, skin pores, spots and texture before and after treatment. (*p < 0.05 vs. Baseline.).

Baseline skin pores, texture, wrinkles and spots, which were assessed using the VISIA, were compared with the follow-up measurements and are presented in Figure 2. In comparison to baseline, wrinkles improved as early as one month and remained improved at follow-up. After two treatment, skin pores have a statistically significant improvement comparing with baseline but not in spots and texture. For spots, a trend of improvement at final follow-up were observed while did not reach statistical significance (*p* > 0.05).

### 3.2. Subjective evaluation

All subjects rated their satisfaction for laser treatment on a 0–3 numerical scale at the final follow-up, 14.8% (*n* = 4) of subjects were extremely satisfied with the treatment and 55.6% (*n* = 15) were satisfied with treatment, none of patients reported that they were totally dissatisfied. The average satisfaction score was 2.04 ± 0.80. Majority subjects believed that their skin become better and 44% (12/27) subjects stated their skin looked more radiant and brighter. The change of the skin was consistent with the change trend of the MI in this study. In addition, using a 1–10 pain scale (VAS), subjects evaluated the pain associated with laser treatment, the average pain in the treatment was 3.21 ± 0.60. Similarly, investigator rated GSP score as well as patients' satisfaction were equivalent at the final follow-up mark Table 3.

**Table 3:** Subjective evaluations.

Patient-evaluated outcomes	
Patient satisfaction	2.04 ± 0.80
Mean pain score (SD)*	3.21 ± 0.60
Global score for *p*hotoaging	2.67 ± 0.47 (*p *< 0.001)**

* Rated on a 0–10 point visual analog scale, VAS (0 no pain, no heat sensation; 10 severe pain, severe heat sensation).

** Global Score for Photoaging compared to baseline. The baseline was showed in the Table 1 (3.44 ± 0.50).

### 3.3. Adverse effect

Adverse events were mild and self-limited. Transient side effects included edema and heating, which were immediately noted after all treatments and disappeared within 24 h. In addition, erythema was also noted after all treatments which resolved within one week. Dryness and desquamation were observed after treatment in two patient whose skin hydration were in a low level at baseline. There were no instances of post-inflammatory hyperpigmentation (PIH).

## 4. Discussion

The fractional lasers are widely used in the treatment of photoaging (15, 19). Although the ablative fractional lasers have faster results, they are associated with a higher complication rate, along with longer downtime and greater recovery time. The non-ablative fractional lasers, compare with the ablative fractional lasers, are associated with a low risk of adverse effects and are suitable for nearly any patient, while repeated treatments could be required to achieve the desired results.

In this prospective study, the FTL is being evaluated for its safety and efficacy for treating multiple signs of photoaging in Chinese adults. More than 70% of the subjects were satisfied or extremely satisfied with the treatment effect of the FTL after three consecutive treatments spaced one month apart.

**Figure 3:**
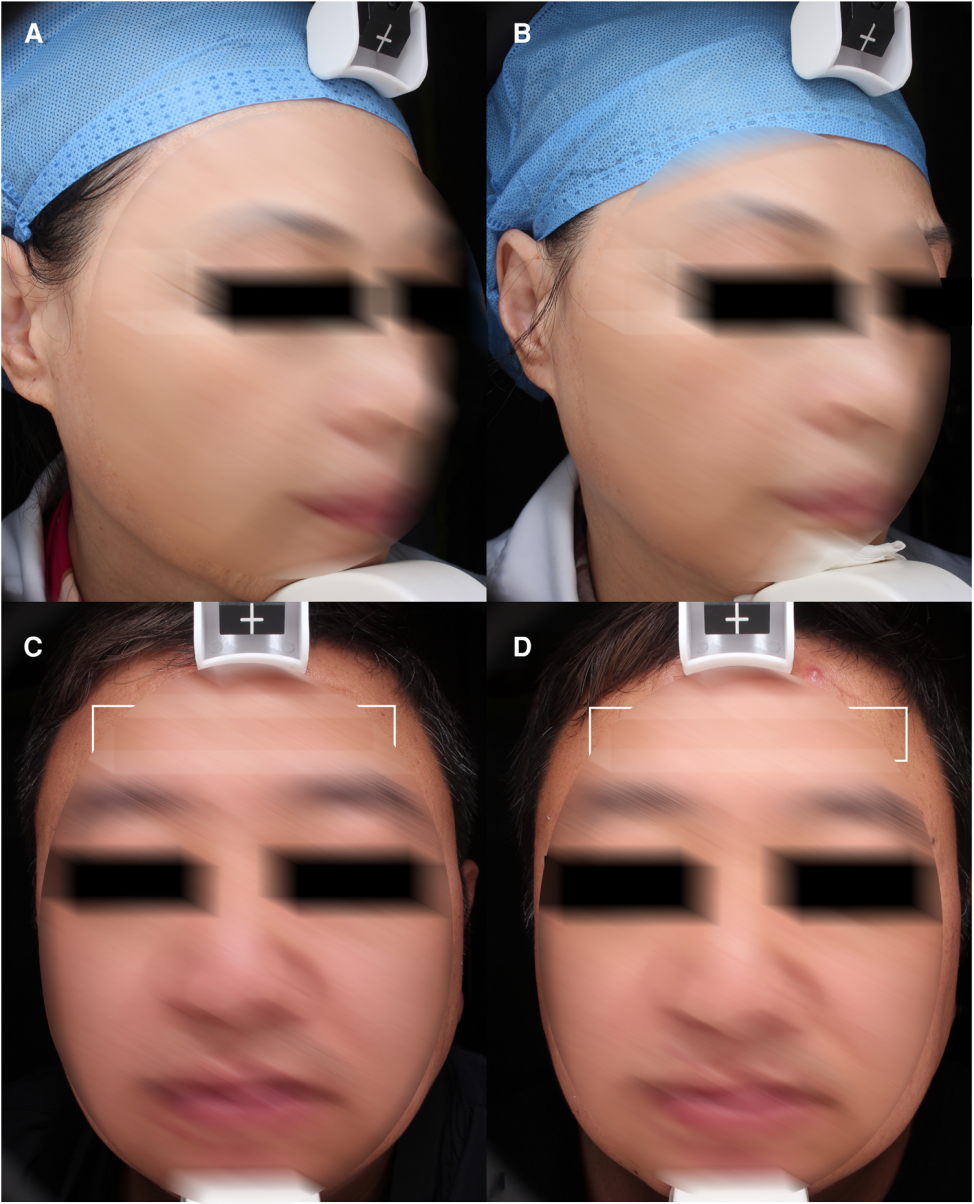
Wrinkles. (**A**) and (**C**) baseline, (**B**) and (**D**) 1 month after third treatment (1927 nm, 6 w, 700 us, 2 passes).

The first study of South Korean researchers utilizing FTL for treatment of photoaged demonstrated promising results in Asian skin (20). Afterward, Wu et al. reckoned that FTL is more effective in the treatment of wrinkles and relaxation than intense pulsed light or Nd: YAG 1064/532 nm laser (21, 22). Skin retraction time was used as a marker of skin elasticity in this study. The reduce of skin retraction time means the improvement of skin retraction. The results suggest that the skin retraction time was significantly reduced compared with the baseline after one month of all treatments. Furthermore, it could be found that the meaning of the wrinkle index was closely related to the age of the subjects. According to the wrinkle data identified by VISIA, there was statistically significant improvement in the subjects (Figure 3). Though the dermal density of some subjects was denser than before, there was no significant improvement in the data. It might be related to the limited sample size and the accuracy of machine measurement.

**Figure 4:**
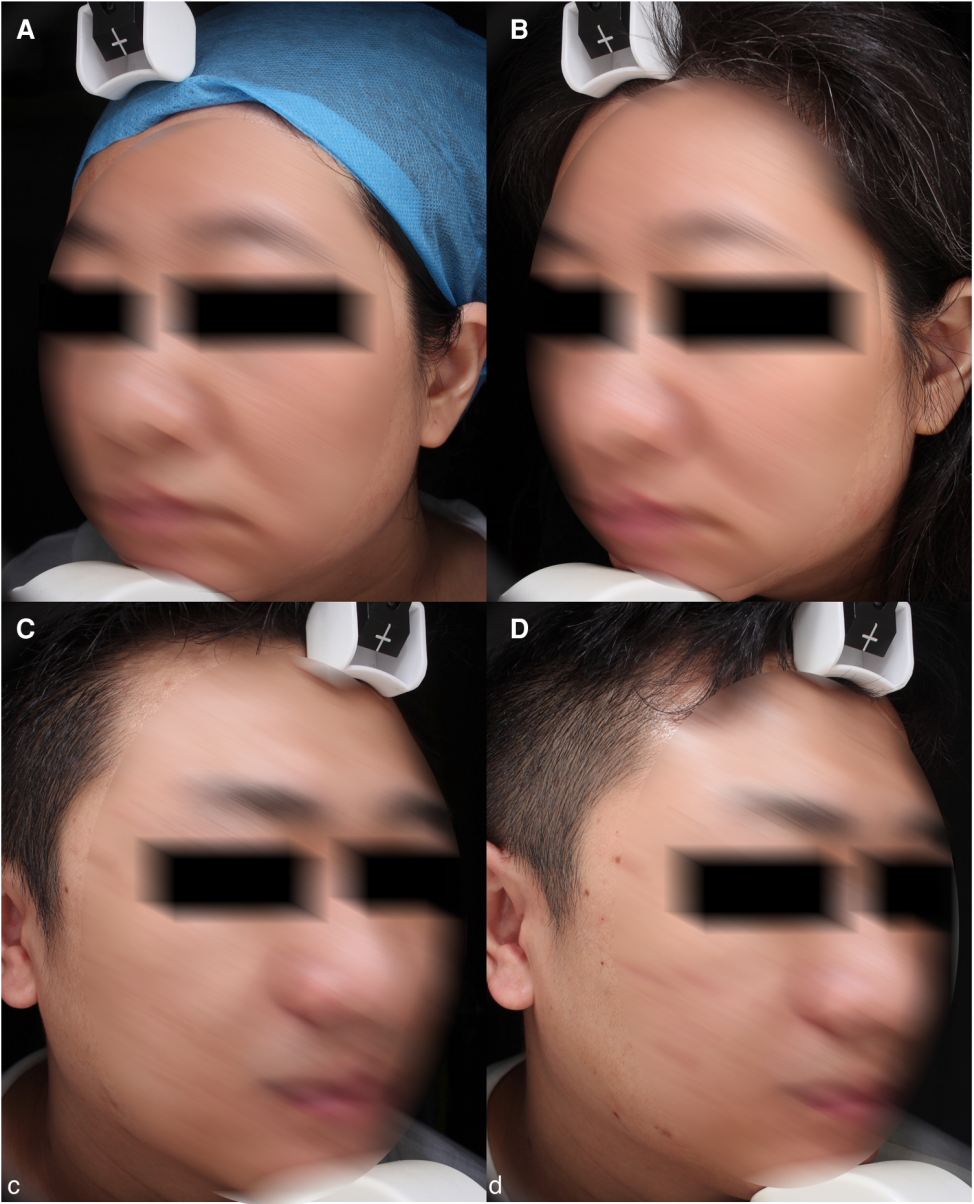
Pores. (**A**) and (**C**) baseline, (**B**) and (**D**) 1 month after third treatment (1927 nm, 7 w, 800 us, 2 passes).

This trial shows a significant improvement of enlarged pores among the photoaging symptoms after one month of all treatments (Figure 4). Although the data is not precise enough, some subjects showed significant improvement in scar of acne in photograph, which is similar to Lu et al. (17).

**Figure 5:**
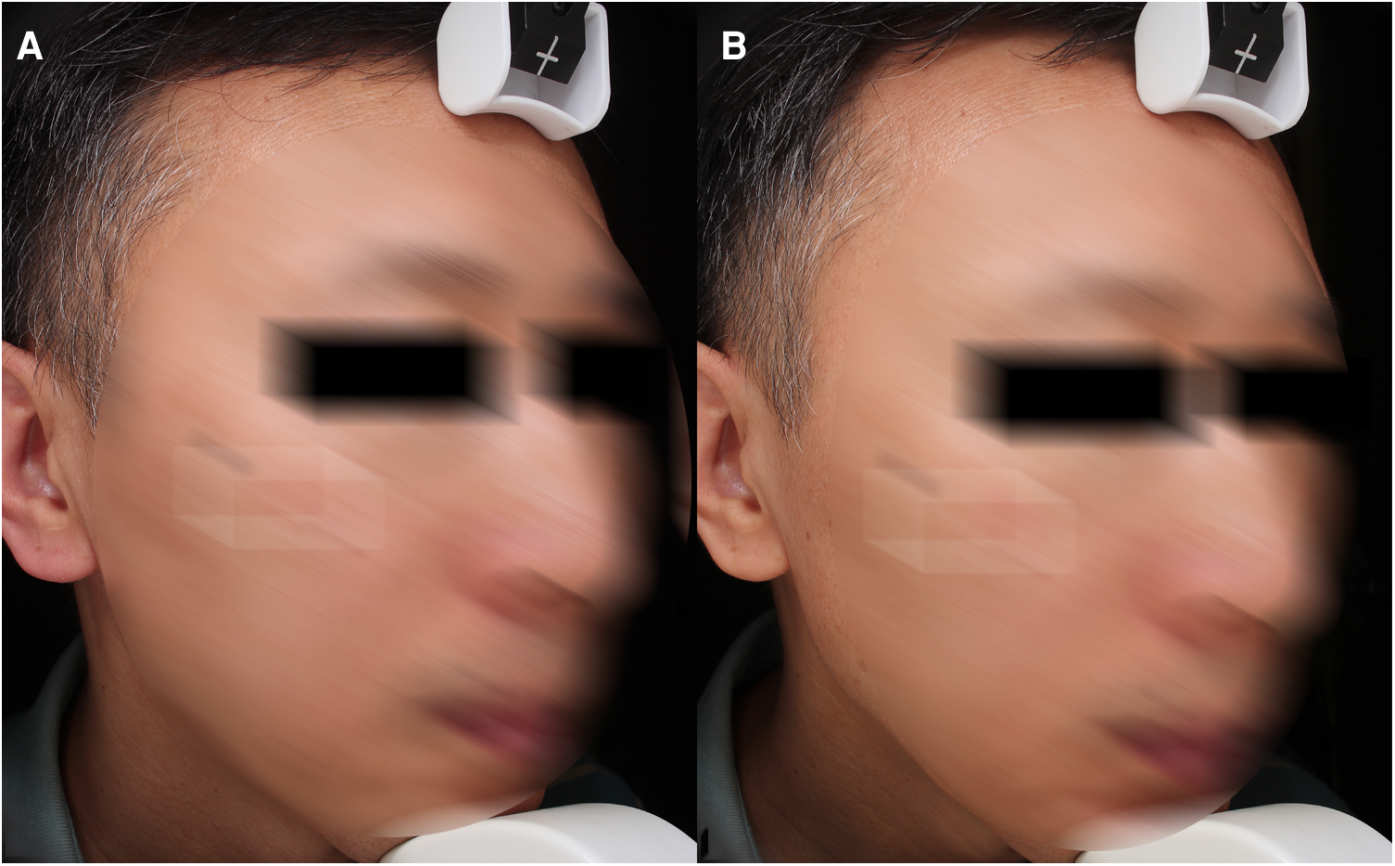
PIH and pores. (**A**) baseline, (**B**) 1 month after third treatment (1927 nm, 7 w, 800 us, 2 passes).

**Figure 6:**
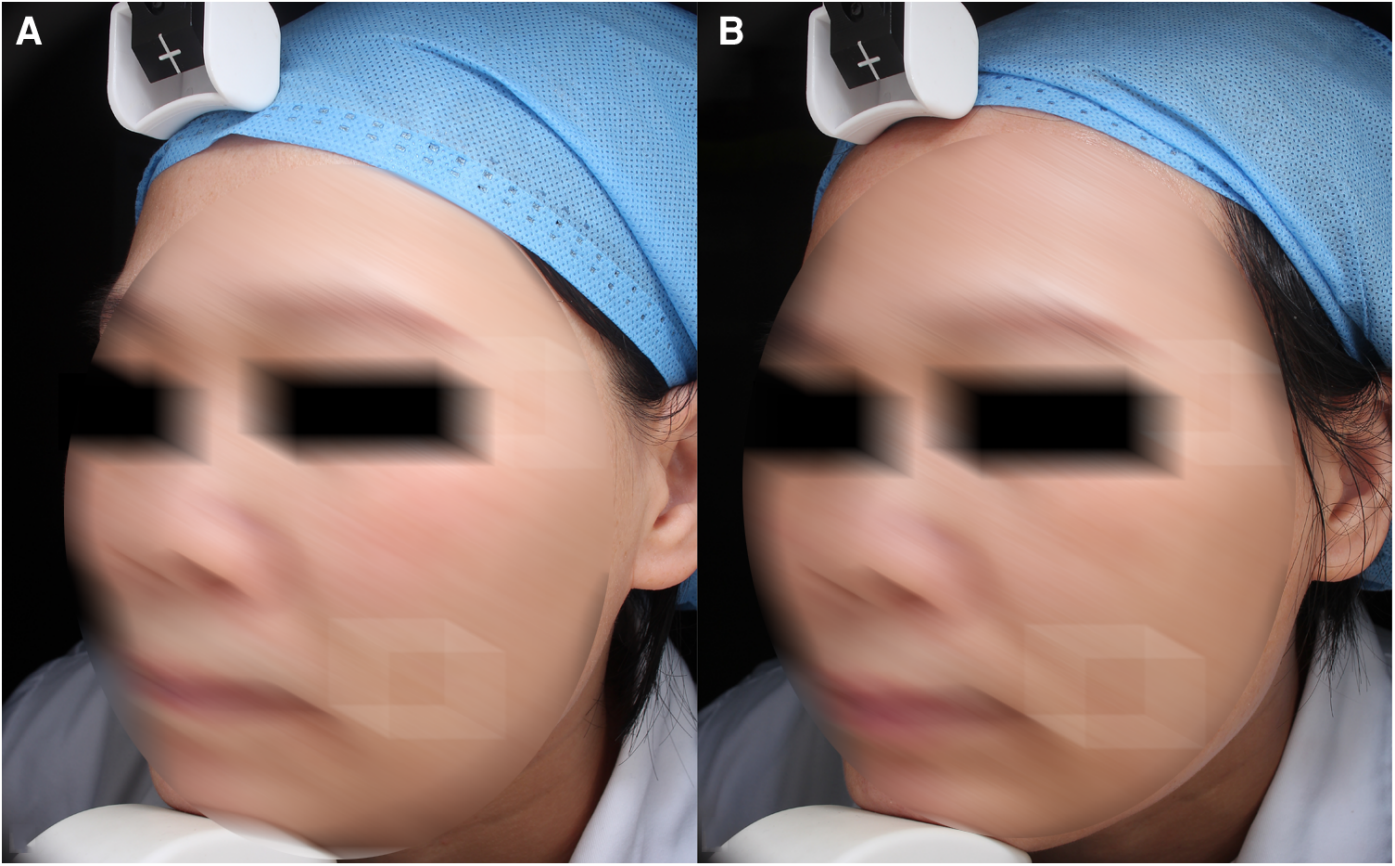
PIH. (**A**) baseline, (**B**) 1 month after third treatment (1927 nm, 6 w, 600 us, 1 passe).

After Lee et al. used FTL to treat Asian melasma in 2013 (20), more and more studies on treating post-inflammatory pigmentation by FTL have also been reported. After Bae et al. (23) first uses FTL to treat PIH treatment, Alharbi found it was more friendly for patients with darker skin types (24). In addition, due to its possibly increased safety profile over other fractional lasers, FTL appears to have a significant role to play in the treatment of skin of color. There could be a unique mechanism of action that explains this increased safety profile. The FTL has a moderate affinity for water-containing tissues, which prevents epidermis turnover rather than causing it. Nevertheless, it is able to penetrate deep into 200–300 μm and stimulate modest collagen regeneration (25). In this study, MI was significantly improved from baseline, which was consistent with the subjective expression of skin lightening in 44.4% subjects. Standardized photos of some subjects showed that PIH was better than before (Figure 5). However, the spots did not see obvious difference. It remains difficult to ensure accurate measurement of in photoaging patients because of their complex and mixed facial situations, including chloasma, PIH, facial pigmentation disorder, freckles, the pigmented nevus and seborrheic keratosis etc (Figure 6). The inclusion of all hyperpigmented diseases resulted in no statistically significant difference in the results.

Both intrinsic and extrinsic aging could lead to thinning of the epidermis, which Pena and his colleagues demonstrated using multiphoton multiparametric 3D quantification tools (2). This study showed an increase in epidermal thickness after three treatments, indicating that FTL could reverse skin aging to some extent.

There are some potential limitations of this work. First, there is no control arm and a small sample size. Therefore, the improvement of different Fitzpatrick skin types before and after the treatment separately, which might enrich the experimental results, may be present in the future study. Secondly, there is no follow-up period for observing long-term effects, such as six months or even a year. Clinical trials result in observations with varied parameters. To overcome these limitations and to provide stronger clinical recommendation, enlarged samples of patient as well as the use of comparative device trials or split-face trials are required.

## 5. Conclusion

The use of lasers for photoaging in Chinese patients has been studied in a limited number of studies. Photoaging patients with wrinkles, large pores and yellowish skin benefit from FTL treatment in this study. To better understand FTL's mechanism and maximize its effect, more well-designed clinical trials with higher sample sizes and histopathology analyses are needed.

## Data Availability

The raw data supporting the conclusions of this article will be made available by the authors, without undue reservation.
